# Primary Care Informatics: Vitalizing the Bedrock of Health Care

**DOI:** 10.2196/60081

**Published:** 2024-10-15

**Authors:** Jacqueline Guan-Ting You, Tiffany I Leung, Deepti Pandita, Matthew Sakumoto

**Affiliations:** 1 Clinical Informatics Mass General Brigham Somerville, MA United States; 2 Department of Medicine Brigham and Women's Hospital Boston, MA United States; 3 Department of Internal Medicine (adjunct) Southern Illinois University School of Medicine Springfield, IL United States; 4 JMIR Publications, Inc. Toronto, ON Canada; 5 Department of Medicine University of California Irvine Irvine, CA United States; 6 Department of Medicine University of California San Francisco San Francisco, CA United States

**Keywords:** health care delivery, primary care, primary health care, primary prevention, quality of health care, holistic care, holistic medicine, people-centric care, person-centric care, medical informatics applications, primary care informatics, medical informatics, health informatics, information science, data science

## Abstract

Primary care informatics (PCI) professionals address workflow and technology solutions in a wide spectrum of health, ranging from optimizing the experience of the individual patient in the clinic room to supporting the health of populations and augmenting the work of frontline primary care clinical teams. PCI overlaps uniquely with 2 disciplines with an impact on societal health—primary care and health informatics. Primary care is a gateway to health care access and aims to synthesize and coordinate numerous, complex elements of patients’ health and medical care in a holistic manner. However, over the past 25 years, primary care has become a specialty in crisis: in a post–COVID-19 world, workforce shortages, clinician burnout, and continuing challenges in health care access all contribute to difficulties in sustaining primary care. Informatics professionals are poised to change this trajectory. In this viewpoint, we aim to inform readers of the discipline of PCI and its importance in the design, support, and maintenance of essential primary care services. Although this work focuses on primary care in the United States, which includes general internal medicine, family medicine, and pediatrics (and depending on definition, includes specialties such as obstetrics and gynecology), many of the principles outlined can also be applied to comparable health care services and settings in other countries. We highlight (1) common global challenges in primary care, (2) recent trends in the evolution of PCI (personalized medicine, population health, social drivers of health, and team-based care), and (3) opportunities to move forward PCI with current and emerging technologies using the 4Cs of primary care framework. In summary, PCI offers important contributions to health care and the informatics field, and there are many opportunities for informatics professionals to enhance the primary care experience for patients, families, and their care teams.

## Introduction

### Introduction to Primary Care

Primary care is defined by the World Health Organization as “a model of care that supports first-contact, accessible, continuous, comprehensive and coordinated person-focused care” while achieving equitable access to care and emphasizing population health [1]. Primary care is one of the only sections of health care shown to have positive impacts on health outcomes and population health, emphasizing its fundamental role in the health and well-being of the public [2]. Primary care specialties can include internal medicine, family medicine, and pediatrics [3,4]; some definitions also include obstetrics and gynecology [5,6].

Primary care is the backbone of health care and is crucial to the health of populations. One international survey indicates that about 75% of respondents had a usual source of primary care [7]. Recent US data indicate that 55% of public insurance (Medicaid) beneficiaries primarily visited primary care clinicians and 36% of patients predominantly visited specialists [8]. Having a usual source of care, such as primary care, is associated with increased preventive screenings such as blood pressure measurements and diabetes and cholesterol screening [7]. Access to primary care has been correlated with lower avoidable hospitalization rates [9]. Loss of primary care has been associated with increased utilization of specialty, urgent care, and emergency care among older adults [10].

Stange et al [11] refer to primary care as an essential bridge between the health of individuals and population health, “situated at the unsettled boundary region between the messy but meaningful lives of individuals, families, and communities, and the health care, public health, and social systems designed to support them.” Areas where primary care can collaborate with public health and play a role in population health include but are not limited to chronic disease management, communicable diseases, and maternal-child health [12]. This role as a mediator also includes facilitating care with medical specialists and social services [13]. Because of its unique position in facilitating numerous interactions between different parties, primary care serves as a central hub for various data streams and care workflows; informatics solutions in this discipline therefore must match the field’s unique needs.

### Introduction to Primary Care Informatics

Primary care informatics (PCI) augments the crucial work that primary care provides to populations. In 2003, the Primary Care Informatics Working Group under the American Medical Informatics Association defined PCI as the “application of information technology to improve the practice, education, and research of primary care, and the development of informatics tools appropriate for primary care practice” [14]. At the time, PCI was emerging globally to address the use of technology in areas such as chronic disease management. de Lusignan [15] argued that PCI was not just a discipline of technology but rather a “science” dedicated to the promotion of “patient-centered primary medical care” with a biopsychosocial model of health; PCI was distinct from other areas of health informatics due to the use of heuristics-based clinical management and patient-centered consultations. The Primary Care Informatics Working Group of the International Medical Informatics Association was formed in the early 2000s [15], with the aims of addressing real-world primary care data and ontologies for research; technology integration in primary care; and ethical, legal, and social issues related to primary care [16].

PCI professionals bring unique expertise at the nexus of primary care and informatics, well before the COVID-19 pandemic. We define PCI professionals as inclusive of clinicians, researchers, technology professionals, experts, and others who engage in PCI. A few examples of PCI issues included the impact of electronic health records (EHRs) on primary care [17-19], benefits of health information exchanges in primary care [20], evidence on health care workers’ perceptions of mobile health in primary care [21], and risks and benefits of artificial intelligence (AI) in primary care [22]. The COVID-19 pandemic put a spotlight on the central role of primary care clinicians globally to mobilize and continue to provide access to care and public health care services to populations all over the world during lockdowns and additional restrictions. PCI professionals rapidly deployed and scaled up telehealth visits and identified and addressed inequities in the digital divide (such as those due to internet access) [23]. Telehealth and telemedicine positively impacted primary care by providing improvements in key areas including continuity, accessibility, care coordination, comprehensiveness, access, better appointment times, and efficiency [24]. In response to the COVID-19 pandemic, additional opportunities were highlighted to further strengthen systems, including vaccination and disease prevention, disease management, public health surveillance, and pandemic preparedness [25].

In this viewpoint paper, we highlight issues uniquely serviced by PCI, distinct from health informatics as a whole, discussing aspects of PCI that have evolved since the early 2000s to mark the 25th anniversary of *JMIR Publications* and the *Journal of Medical Internet Research*. In particular, in the wake of COVID-19, there have been changes to primary care practice pertinent to PCI, including challenges to clinician well-being and digital health transformation in domains such as telehealth [26].

## Common Challenges in Primary Care

Challenges may vary for different regions of the same country; for example, in the United States, access to primary care for adults [27,28] and pediatric patients [28] is more challenging in rural than urban settings. Differences in primary care worldwide range from physician consultation time [29] to funding and structure [30]. Countries and geographic regions have different needs in primary care—for example, Japan has universal health care and an aging population with challenges around chronic disease management [31], while in sub-Saharan Africa, the disease burden consists of both chronic diseases as well as infectious diseases [32]. In many primary care practices, unlike most specialties, outcomes are addressed at both the individual patient and population levels; at times, these perspectives are not always aligned, for example, when individual values or beliefs may differ from the population or public health recommendations, or when guidelines do not fit a patient’s clinical presentation or health conditions. Yet, the responsibility of addressing these differences in shared decision-making can fall to primary care clinicians to reconcile with patients.

A recent report on US primary care identified the lack of usability and interoperability of digital health tools as barriers to health care organizations and clinicians unlocking the full possibilities of health information technology—“real potentials of digital health to aggregate a wide array of medical, environmental, biological, and social data; make meaningful sense of information; automate care; make care proactive and not reactive; enhance health equity; and enable population health monitoring and management have been barely explored” [2]. Similarly, recent international physician survey data suggest the majority of primary care physicians are not able to adequately coordinate care with specialists and hospitals [33], in part due to poor data access and communication, a gap that PCI professionals can help address.

Also, there are troubling themes in recent primary care challenges that cross borders, such as clinician burnout [34], aging populations [31], chronic disease prevalence [32], significant administrative work [35], and mismatch of patient demand and clinician supply [32], which in turn threaten the health of communities globally. However, the authors are optimistic that greater awareness of the opportunities and changes possible through intentional applications of PCI, building on the last 25 years of evidence-based advancements and development in the field, can pave the way forward for continued primary care, and consequently, overall health system improvements. The authors share perspectives reflecting their current and prior primary care experience in the United States, Europe, and Asia, although they expect that some themes described may be applicable to certain health care systems and settings elsewhere.

## Evolution of PCI

### Overview

PCI has evolved to address some of these new challenges. de Lusignan [15] highlighted 3 core areas of PCI, including heuristic decision-making, patient-centered care, and the biopsychosocial model. Building on the principles de Lusignan [15] highlighted, recent trends in primary care with PCI at its core include personalized medicine, analytics- and data-driven population health, social drivers of health, and team-based care. Heuristic-based care is now bolstered by point-of-care analytics and data-driven decisions derived from population health analytics; patient-centered care is informed by personalized medicine; and the biopsychosocial model is strengthened by improved awareness and screening of social determinants. The planted seeds of PCI in the early 2000s have grown and flourished with new technologies and new models of primary care.

### Personalized Medicine, Augmented Personalized Decision-Making, and Customization of Care

Primary care clinicians often engage in shared decision-making—described in one model as a four-step process of (1) recognizing the opportunity to make a decision with patient and clinician, (2) introducing a decision aid, (3) having a conversation, and (4) receiving care [36]. This decision-making allows for more personalized and arguably patient-centered care. Personalized medicine has the potential to provide new resources and tools to facilitate shared decision-making.

The growth of genetics knowledge has offered new opportunities to consider how genomic medicine may potentially be integrated into primary care—for example, a real-world pilot with large gene sequencing panels in primary care [37] versus the integration of genetics counselors into primary care teams [38]. Furthermore, the nexus of precision medicine and AI will enable improved care—such as more precise medication dosing, improved treatment recommendations based on comorbidities, or improved risk prediction for diseases [39]—areas in which PCI professionals can inform research and subsequent practical implementation. AI also has the potential to blur the lines between personalized medicine and population health—for example, with precision population analytics, creating “precision cohorts” similar to a primary care clinician’s patients can help better inform which treatment option to use based on population-level data [40].

Digital health tools, such as wearables and mobile health apps, can provide patient-centered insights and shift primary care toward proactive yet targeted “precision prevention” [41]. These technologies can drive customized care with shared decision-making and allow primary care to pivot toward primary prevention and not just chronic disease management. Along with the personalized treatment of health conditions, patients are better able to customize their care experience; patients are more empowered to access care flexibly outside of brick-and-mortar clinics through mediums such as telehealth [42] and patient portal messages [43].

### Population-Based Approach Driven by Big Data and Analytics

Primary care is uniquely positioned to become a potential hub for better management of the vast amounts of unused health care data across clinical settings. Each hospital produces an average of 50 petabytes of data annually with an estimated 97% of data remaining unused [44]. EHRs, wearables, and other technologies have expanded modalities [45] and the volume of health data [46]. Health information exchanges can augment the data of individual systems, powering descriptive, predictive, and prescriptive analytics to facilitate quality, improved risk management and clinical decision-making at the population level [47]. Primary care, where longitudinal chronic care and multidisease management take place, can be a vital central hub for patient and population health data. Wagner’s [48] widely adopted chronic care model includes information systems as a core component of providing high-quality chronic disease care. Data-driven improvement using computer-based systems is also considered a building block for a high-performing primary care practice [49]. While extensive research on predictive machine learning has been performed in areas such as cardiac disease, mental health, communicable diseases, and perinatal outcomes, work needs to be done to take into account model usability in the real world [50], as well as practical considerations such as external validation for pragmatic clinical decision-making [51].

Furthermore, as technologies, like generative AI, are implemented across health care [52,53], PCI professionals have and continue to lead the charge in steering the primary care and informatics communities toward equitable [54-56] and ethical [57] approaches to implementing these technologies. For example, experts spanning PCI, policy, bioethics, and philosophy for the largest US medical specialty organization [58] recently called for ethical, accountable, equitable, transparent, and environmentally aware use of AI in health care [59]. PCI professionals can advocate for appropriate safeguards balanced with clinically reasonable regulations to avoid some of the pitfalls associated with current EHR administrative work [60].

Population health can encompass chronic disease management—examples of PCI initiatives in this area include chronic care management and remote patient monitoring programs, which provide continuous support across the care continuum [61]. Remote patient monitoring is most often applied in chronic disease management for cardiology, endocrinology, pulmonary, and geriatrics care [62]; beyond expanding the interface modalities and underlying AI models [63] powering this technology, PCI can add value by improving the patient and clinician experience with these technologies [64]. Even as analytics and associated technologies for addressing population health continue to evolve, PCI professionals can help ground these technologies in a sociotechnical design or implementation approach with appropriate outcome measures (such as patient-reported outcome measures) [65].

### Social Drivers of Health

As part of comprehensive, culturally sensitive, and patient-centered care, PCI professionals address social determinants of health (SDOH), also referred to as social drivers of health, in EHR design or improvements [66-68]. SDOH includes areas such as education, food, housing, income, and other nonmedical factors that contribute to health [69]. Recent attention to these drivers has advanced the traditional biopsychosocial model of care. The longitudinal relationship of patients with primary care clinicians, as opposed to the episodic nature of other specialties, allows for greater patient trust in disclosing SDOH information during primary care visits. Documenting and visualizing SDOH gaps increases the likelihood of addressing these factors that greatly impact patients’ well-being [70]. PCI professionals can identify and present resources to both patient and clinician, matching the right intervention with the right patient to maximize benefit to the patient and community.

Other examples of relevant determinants within SDOH also include commercial determinants of health (industry entities shaping health choices in areas such as food, alcohol, tobacco use, pharmaceuticals, and media consumption) [71,72]; political determinants of health (sociopolitical events correlating with disparities in health outcomes) [73]; and geographic location (using geographical information systems to measure disease incidence and prevalence, health risk, community access to health services, and community health profiling) [74]. In addition, health informatics researchers and PCI professionals play a role in addressing immigrant and refugee health, for example, in building appropriate record systems to accommodate the international needs of this vulnerable population [75]. There is a significant intersection between PCI and the emerging field of social informatics [66], which deals with data from a breadth of sources to advance social and medical care. Furthermore, as PCI implements patient-facing solutions (eg, in digital health), the field should aim for meaningful, equitable solutions built on community engagement [76], which can allow for culturally relevant informatics tools that engage and advocate for underrepresented populations [77].

### Informatics to Enable Dynamic, Flexible Team-Based Care

Primary care teams can vary in composition but can include nurses, medical assistants, health coaches, behavioral health specialists, and pharmacists supporting primary care providers, often coupled with clinical workflows such as standing orders or protocols [78]. There is evidence that these team members can provide effective care in particular areas of primary care; for example, primary care nurses provide effective care in areas such as chronic disease management, care management, and health promotion [79]. Team-based primary care has been correlated with improved quality measures, such as depression and diabetes care adherence, and reduced emergency department visits and hospitalizations [80]. This team-based care is a key component of care management, supporting higher-risk patients with better disease management and care navigation; care management in turn is vital to promoting value-based-care—care that is patient-centered and improves clinical outcomes while avoiding unnecessary costs [81].

Health information technology has been shown to be an important part of effective team-based primary care. [82] For example, the advanced team-based care model integrates technology into a care team model with enhanced clinical workflows and roles, including a care team coordinator whose responsibilities include history taking during clinic visits, coordination of preventive health screenings, clinician-supervised laboratory test or order placement and documentation; this model improves patient outcomes such as cervical cancer screening and immunizations and allows physicians or advanced practice providers to focus on communication, clinical decision-making, and management of a greater number of health issues [83]. PCI professionals also can introduce new tools to team-based care such as secure messaging for interprofessional communication [84,85]. With increases in telehealth since the COVID-19 pandemic, PCI professionals have been core to establishing new care team models specific to telehealth, taking into account teams that are often remotely located [86].

## Opportunities in PCI

### Overview

A broad range of digital health interventions for prevention in primary care exist, ranging from EHR-based solutions to telehealth, that have proven efficacy but have not been widely adopted or are primarily clinician facing [87,88]. We break down current and future state technologies that have the potential to transform the primary care experience.

We apply the 4Cs of primary care (alternatively known as the 4 tenets or 4 pillars of primary care) framework [89,90] to different domains and tasks encountered in primary care and map these domains and tasks to health informatics tools (Table 1). Some of these domains may encompass multiple pillars.

**Table 1:** 4Cs of primary care, primary care domains and tasks, and corresponding informatics tools or aspects.

4 Cs of primary care and primary care domain or task		Health informatics tools or aspects
**Contact**		
	Patient engagement and patient education	Mobile health apps, text messaging, telehealth, medical extended reality, personal health records, patient portals
**Comprehensiveness**		
	Chronic disease management	Remote patient monitoring, EHR^a^ design, telehealth visits, mobile health
	Population health management	Clinical decision support, machine learning analytics, disease registries
	Social determinants of health	Clinical decision support, EHR design
**Coordination**		
	Care team or clinician-clinician coordination	Interoperability, EHR design, electronic consults
	Documentation or administrative tasks	Ambient listening documentation, generative AI^b^ chart summarization
	Population health management	Precision population analytics, predictive analytics, health information exchange
	Collaboration with public health	Data aggregation, interoperability, data reporting, learning health systems
**Continuity**		
	Patient-clinician communication	Patient portal messaging, telehealth

^a^EHR: electronic health record.

^b^AI: artificial intelligence.

### Contact

The first C, contact, refers to patients accessing primary care services. With the necessitated movement to telemedicine and telehealth services during the COVID-19 pandemic, telehealth adoption among certain patients was rapid [91]. After the pandemic, there is much work to be done to address gaps in telehealth access, especially among those of lower socioeconomic status [42].

Health care systems must rethink how individuals access health care, and PCI practitioners are poised to bridge gaps in access and creatively engage patients in care. Text messaging for medication adherence in chronic diseases [92] and mobile health have engaged patients with chronic health conditions [93,94]. On the other hand, medical extended reality is a growing field overlapping with clinical informatics; its applications span from behavioral health to integration with procedural specialties [95]. Patient education is a key component of primary care visits; virtual reality has been studied for patient education [96], and in the future, could be a tool in PCI for patient education and engagement. Deep learning can also improve patient instructions by “translating” language to an appropriate reading or health literacy level [97]. While benchmarking suggests large language models’ multilingual functions do not perform well enough compared to commercial translation services [98], large language models could someday create high-quality multilingual patient education materials.

### Comprehensiveness

The second C, comprehensiveness, refers to patient-centered management of the breadth of conditions and a balance of prevention and treatment.

To provide comprehensive care, primary care clinicians and other care team members must be able to find the right information for the right patient (or even groups of patients) at the right time. Interoperable and usable EHRs are key to timely and appropriate data access. However, current EHR usability is often poor and associated with clinician burnout [99]. Similarly, a recent survey of US family physicians demonstrates poor satisfaction with interoperability in primary care [100]. PCI professionals, in collaboration with experts in these domains, can help facilitate improved clinical workflows, data infrastructure, and user-friendly designs to advance comprehensive care.

PCI professionals can also augment population health management, where clinical decision support has been shown to positively impact screenings for cancer, infectious diseases, and cardiovascular risk factors [101]. Health information management systems through health information exchanges have been associated with better care outcomes [102]; data analytics in primary care are correlated with decreased emergency department visits and increased care engagement [103]. Disease registries, such as those for chronic kidney disease [104], can assist primary care teams in better managing chronic diseases at a population level. Other digital health interventions can help with preventive care, such as immunization and cancer screening, and improve clinical outcomes [87]. As mentioned earlier, the integration of social drivers of health into informatics tools and clinical workflows will also improve patient-centered care.

### Coordination

The third C, coordination, refers to bringing together different parts of the health care system and serving as a bridge between primary care and specialty care. Primary care serves as a contact point to other parts of the health system, including medical specialists and social services.

Care coordination has been positively correlated with improved care measures such as diabetes, cardiovascular, and cancer screening [105]. Electronic consults (eConsults) thus far have had mixed outcomes [106] but still have the potential to assist with coordination between primary care and specialists and address access to specialty care.

Care coordination is inextricably linked with communication. Interoperability and facile interfaces for communication can improve interactions between different care team members, and when coupled with timely and convenient access to data, are powerful in advancing patient care. National learning health systems have been posited as a framework to advance interoperability and digital health innovation [107] for the public good.

Coordination includes administrative tasks around patient care such as documentation of patient encounters. Generative AI solutions, such as ambient AI [108] and medical record summarization [109], have the potential to alleviate the documentation burden in this area and lead to more concise clinician-clinician communication.

### Continuity

The fourth C, continuity, has highly variable definitions, especially in the context of team-based care, but tends to refer to the patient’s ability to receive care that is centered around relationships and has a sense of longitudinality. Primary care continuity correlates with decreased patient mortality [110]. As mentioned previously, telehealth has provided new touchpoints for primary care practitioners and their patients. Patient portals [43] and patient access to notes [111] have both been shown to increase patients’ understanding of their health, which arguably in turn can be an opportunity for primary care teams to engage patients in their health over time and build trust with patients. Continuity can also be extended to transitions of care between care settings that intersect with primary care, such as inpatient hospitalization, where there is a current dearth of patient-clinician communication tools [112]—this is an area where PCI can provide new bridges for continuity.

## Discussion

Primary care is the bedrock of health care and PCI in turn can vitalize this bedrock—contributing to societal health and well-being by improving existing workflows or thoughtfully integrating new technologies into care. PCI melds the learnings of primary care and informatics but is also able to add distinctive value to both fields by augmenting the core tenets of primary care with biopsychosocial-driven technologies. At a time when the global primary care workforce is facing immense challenges, PCI professionals have a key role to play in supporting their primary care colleagues, disseminating best practices learned in primary care to other informatics colleagues, and adapting appropriate tools from other branches of biomedical and health informatics into primary care. PCI is evolving as care is delivered across a greater spectrum of settings and patients are empowered with new digital health tools to engage in their health. They can benefit from new opportunities and tools for proactive preventive care rather than reactive chronic disease management. Ultimately, PCI has been an effective area within primary care and will continue to be critical in driving improved patient and clinician experiences for safe, equitable, effective, patient-centered, and team-based care.

## Abbreviations

AI: artificial intelligence
EHR: electronic health record
PCI: primary care informatics
SDOH: social determinants of health
